# The impact of a fine-scale population stratification on rare variant association test results

**DOI:** 10.1371/journal.pone.0207677

**Published:** 2018-12-06

**Authors:** Elodie Persyn, Richard Redon, Lise Bellanger, Christian Dina

**Affiliations:** 1 INSERM, CNRS, UNIV Nantes, l’institut du thorax, Nantes, France; 2 Department of Medical and Molecular Genetics, King’s College London, London, United Kingdom; 3 CHU Nantes, l’institut du thorax, Nantes, France; 4 Laboratoire de Mathématiques Jean Leray, Nantes, France; McMaster University, CANADA

## Abstract

Population stratification is a well-known confounding factor in both common and rare variant association analyses. Rare variants tend to be more geographically clustered than common variants, because of their more recent origin. However, it is not yet clear if population stratification at a very fine scale (neighboring administrative regions within a country) would lead to statistical bias in rare variant analyses. As the inclusion of convenience controls from external studies is indeed a common procedure, in order to increase the power to detect genetic associations, this problem is important. We studied through simulation the impact of a fine scale population structure on different rare variant association strategies, assessing type I error and power. We showed that principal component analysis (PCA) based methods of adjustment for population stratification adequately corrected type I error inflation at the largest geographical scales, but not at finest scales. We also showed in our simulations that adding controls obviously increased power, but at a considerably lower level when controls were drawn from another population.

## Introduction

Association studies have identified many common variants associated with a wide spectrum of diseases. With the advances in sequencing technologies, it became possible to perform case-control studies on rare variants, which may also play an important role in disease susceptibility. Due to their low frequency in the populations, the statistical analysis presents challenges as these variants must be tested by groups (commonly defined as genes). Many statistical methods have been developed or adapted to test the association between a group of rare variants and a disease status [1]. Each of these methods assumes a different statistical hypothesis and is able to detect association signals depending on the underlying disease mechanisms which are unknown. A first category of tests, called burden tests, consists in aggregating rare variant counts across the gene to sum up the genetic information per individual [2–7]. Another main category of tests, called variance-component tests (or joint tests) [7–10], assumes a heterogeneous group of variants with different effect sizes, and tests the variance of genetic effects.

Population stratification, i.e. the observation of differences in allele frequencies between populations, due to different ancestries, has been shown to be a confounding factor that could lead to many false positive results in rare variant association studies [11–18]. Rare variants are thought to be more recent than common variants. Therefore they are more likely to be geographically localized and contribute to a fine scale ^2^genetic structure. The impact of such a fine genetic structure on association tests results is still poorly investigated. For instance, two studies [16,18] showed that, without population structure adjustment, the analysis of rare variants in simulated European populations could lead to inflation of gene-based test results. However, it has already been shown that a population structure could be identified at even lower geographical scales such as the Western French population, using common variants [19]. Therefore, it is important to know if such geographical structure could lead to false positive results. Indeed, in order to increase the ability to detect disease genes and reduce costs while sequencing more cases as a priority, the use of controls from reference databases is very common in genetic epidemiology studies. However, these controls may be from a different population ancestry than cases and thus create problems of confusion.

Many rare variant association methods exist and their performance varies depending on the genetic scenario. These methods are also influenced differently by population stratification. A higher inflation in variance-component tests (SKAT [9] and C-alpha [8]) than burden tests has been reported by [18], when comparing simulated European populations. Another study [15], also showed that the C-alpha test [8] presents a higher inflation than a burden test, with population stratification. To go further, [18] showed that depending on the joint allelic distribution in two populations, both burden tests or variance-component tests could show higher inflation.

Standard correction approaches, such as adjusting a model for principal components (PCs) representing the genetic structure among individuals, are able to reduce the inflation of rare variant association results but may also fail in specific scenarios [12]. As rare variants may present a different geographical pattern than common variants, the frequency of variants to include in the estimation of the components has also been discussed [12,17]. From these studies, the computation of PCs from common variants is more? efficient, compared to PCs from rare variants, to adjust for a world-wide population structure.

In this paper, we aim to answer two main questions on various rare variant association test strategies: (1) the impact of additional controls with different levels of fine-scale population structure, and (2) the efficiency of PCA correction methods. The impact on association results was assessed in terms of type I error and power through simulations under different genetic scenarios. In our simulations we considered two populations. Cases were drawn from one population and controls were drawn from both populations in varying proportions depending on the simulation scenario. Different geographical levels were set by varying the migration rate between the two populations. We explored these simulated genetic scenarios, to better relate it to real stratification patterns. These analyses would help to better select external controls when performing association analyses on rare variants; or at least warn against potential bias.

## Materials and methods

### Notations

Rare variant association methods test the association between the disease status of *N* individuals and their genotype information for a group of *P* rare variants. Let **X** be the matrix of genotypes with *X*_*ij*_ ∈ {0,1,2} the count of minor alleles for the *i*-th individual and *j*-th variant. Let ***Y*** be the vector of phenotypes with *Y*_*i*_ = 1 if the *i*-th individual is a case, otherwise *Y*_*i*_ = 0. In equation notations, 0 and 1 superscripts denote respectively unaffected and affected persons.

### Rare variant association tests

We compared different association strategies, commonly used in rare variant association studies, such as burden tests and variance component tests. We also took into account the widely used KBAC test [20] which considers multi-locus genotypes. Finally, we applied what we call “position tests”, DoEstRare [21] and PODKAT [22], which are recent approaches taking into account rare variant positions. The different association tests compared in this work are presented in the **Table 1**. For each category of tests, we either selected the most used or/and the most recently developed.

**Table 1:** Rare variant association tests under comparison.

Category	Description of the strategy	Methods
Burden tests	Computation of a genetic score per individual.	CAST [2], Sum test, wSum [3], aSum [6]
KBAC test	Comparison of multi-locus genotypes counts between cases and controls	KBAC [20]
Variance-component tests	Test of the variance of genetic effects.	SKAT [9], SKAT-O [10]
Position tests	Incorporation of rare variant positions in the test statistic.	PODKAT [22], DoEstRare [21]

#### Burden tests

A first category, called burden tests, consists in aggregating rare allele counts across the gene. We used the following burden tests: cohort allelic sum test (CAST) [2,23], sum test (Sum), weighted sum test (wSum) [3], and the adaptive sum test (aSum) [6]. These methods compute a genetic score per individual and test the association between this score with the disease status. We used different burden tests, with subtle hypothesis differences that may impact their statistical behavior with population stratification. In order to better compare them, we formulated burden tests with a logistic regression model:

$$logit(P(Y_{i}=1))=\alpha_{0}+\beta S_{i}$$

with *S*_*i*_ a genetic score for the individual *i* which is a function of rare allele counts *X*_*ij*_,*j* ∈ {1,…,*P*}, and *β* the regression coefficient. The CAST test is said to be a collapsing strategy as the genetic score indicates if an individual carries at least one rare mutation. This score is:

$$S_{CAST_{i}}=I\left(\sum_{j=1}^{P}X_{ij}\geq 1\right)$$

with *I*(.) the indicator function. For tests other than CAST, the genetic score can be written as a weighted sum of allele counts:

$$S_{i}=\sum_{j=1}^{P}w_{j}X_{ij}$$

with *w*_*j*_ the weight for the variant *j*. In the Sum test, each rare variant presents the same weight *w*_*j*_ = 1,*j* ∈ {1,…,*P*}. In the wSum test, weights $w_{j}=\frac{1}{\sqrt{N*{{\hat{MAF}}_{j}}^{0}*(1-{{\hat{MAF}}_{j}}^{0})}},j\in \{1,\ldots ,P\}$ are a function of ${{\hat{MAF}}_{j}}^{0}=\frac{\sum_{i=1}^{N^{0}}X_{ij}+1}{2N^{0}+2}$, the estimated MAF in controls. In the aSum test, *w*_*j*_ = 1 if the *j*-th variant is considered deleterious, and *w*_*j*_ = −1 if the *j*-th variant is considered protective. A single-marker is previously applied to know if a rare variant is classified as protective.

For all these burden tests, we test the null hypothesis *H*_0_:*β* = 0 with a score test [24,25]. The score statistic *U* and its variance *V* are:

$$U=S^{\prime }(Y-\hat{\mu })$$

$$V=\frac{1}{N-1}{\left(Y-\hat{\mu }\right)}^{\prime }(Y-\hat{\mu })*{\left(S-\bar{S}\right)}^{\prime }(S-\bar{S})$$

$\hat{\mu }=logit^{-1}(\alpha_{0}).1_{N}$ is the vector of estimates under the null hypothesis (expression may change with covariates in the model) with **1**_***N***_ = (1,…,1)′, and $\bar{S}=\left(\frac{1}{N}\sum_{i=1}^{N}S_{i}\right).1_{N}$ the vector of average scores. The test statistic is $Q=\frac{U^{2}}{V}$.

#### Variance-component association tests

Variance-component tests consider the variance of genetic effects. These tests have been developed to better identify association signals in a context of variants with different effect sizes and directions in the same gene.

We used sequence kernel association tests (SKAT) [9,10], which are based on the following logistic regression model:

$$logit(P(Y_{i}=1))=\alpha_{0}+\sum_{j=1}^{P}\beta_{j}X_{ij}$$

with *β*_*j*_,*j* ∈ {1,…,*P*}, the regression coefficients for the genetic effects. This model is a linear mixed-effects model with random genetic effects *β*_*j*_ which follow an arbitrary distribution of mean 0 and variance $w_{j}^{2}\tau$. The null hypothesis *H*_0_:*β*_*j*_ = 0,*j* ∈ {1,…,*P*} is then equivalent to *H*_0_:*τ* = 0. Each variant is weighted by *w*_*j*_ to better discriminate causal from neutral variants.

We also used the optimal version of SKAT, called SKAT-O [10], varying the correlation between genetic effects. SKAT assumes that there is no correlation between genetic effects, while SKAT-O aims to identify the optimal correlation between genetic effects. The test statistic for a given correlation parameter *ρ* test is:

$$Q_{\rho }=\left(Y-\hat{\mu }\right)\prime XWR_{\rho }WX\prime (Y-\hat{\mu })$$

with $\hat{\mu }=logit^{-1}(\alpha_{0}).1_{N}$, the vector of estimates under the null hypothesis as previously defined (expression may change with covariates in the model); **W** = diag(w_j_,j ∈ {1,…,*P*}), the weight matrix; and the correlation matrix between genetic effects **R**_**ρ**_ = (1 − *ρ*)**I**_***P***_ + *ρ***1**_***P***_**1**_***P***_′ with **I**_***P***_ identity matrix of order *p*. For the SKAT test, *ρ* = 0. For the SKAT-O statistic, *ρ* varies between 0 and 1 with a bin of 0.1. The distribution of each test statistic under the null hypothesis is approximated and is described by [26].

#### KBAC test

The kernel-based adaptive cluster (KBAC) test [20] aims to better discriminate causal multi-site genotypes from noise with the use of adaptive weights. Multi-site genotypes are the vectors of individual genotypes ***X***_***i***_,*i* ∈ {1,…,*N*}. Let assume *L* + 1 distinct multi-genotype sites ***X***_**0**_,***X***_**1**_,…,***X***_***l***_,…,***X***_***L***_ we observe in the dataset **X**. The KBAC statistic compares the proportions of these multi-site genotypes in cases and controls, and is equal to

$$KBAC={\left(\sum_{l=0}^{L}w_{l}(\frac{n_{l}^{1}}{N^{1}}-\frac{n_{l}^{0}}{N^{0}})\right)}^{2}$$

with *w*_*l*_ the weight for *l*-th multi-site genotype; $n_{l}^{1}$ and $n_{l}^{0}$ the observed counts of the *l*-th multi-site genotype in cases and controls. Weights are computed adaptively with the choice of a kernel function; greater weights are attributed to genotypes that are enriched in cases. In this paper, we use the hypergeometric kernel, which is the most often used as it is suitable for small to moderate sample sizes. The weight *w*_*l*_ for the *l*-th multi-site genotype is then defined as

$$w_{l}=P(N_{l}^{1}\geq n_{l}^{1})=\sum_{k=0}^{n_{l}^{A}}\frac{\left(\begin{array}{c} N_{l}\\ k \end{array})(\begin{array}{c} N-N_{l}\\ N^{1}-k \end{array}\right)}{\left(\begin{array}{c} N\\ N^{1} \end{array}\right)}$$

#### Position tests

We also investigated the impact of population structure on two statistical strategies which extend the rare variant tests to situations where the position of polymorphisms has an impact in the disease. These two tests are DoEstRare, developed in our team and which tests variant density in cases and controls and PODKAT which extends SKAT through integration of a position distance matrix.

The test PODKAT [22] is an extension of the test SKAT. The test statistic is similar to the SKAT statistic:

$$Q_{PODKAT}=\left(Y-\hat{\mu }\right)\prime XWAA\prime W\prime X\prime (Y-\hat{\mu })$$

with a position-dependent matrix **A** measuring proximities between variants. The proximity measure between variants *j* and *j*′ is:

$$A_{j,j^{\prime }}=\max\left(1-\frac{1}{w}d_{j,j^{\prime }},0\right)$$

with *d*_*j*,*j*′_, the physical distance between variants *j* and *j*′; and the parameter *w* is called maximal radius of tolerance, by default its value is 1,000 bp.

The significance of the PODKAT statistic is based on the approximation of the distribution under the null hypothesis with the Davies’ method [27]. The integration of position-dependent matrix is a strategy that has also been used by [28,29].

The DoEstRare test [21] compares both position distributions on the gene and overall allele frequencies between cases and controls. Let ${\hat{f}}^{1}$ and ${\hat{f}}^{0}$, be the kernel density estimators of position distributions; ${\hat{p}}^{1}$ and ${\hat{p}}^{0}$, the weighted average allele frequencies in cases and controls. The test statistic is:

$$STAT=\int_{1}^{Lg}|{\hat{p}}^{1}*{\hat{f}}^{1}(pos)-{\hat{p}}^{0}*{\hat{f}}^{0}(pos)|dpos$$

with *Lg* the length of the tested region in bp.

Concretely, this statistic corresponds to the area between the density function curves, each multiplied by allele frequencies in cases and controls.

The position density functions are estimated with a Gaussian kernel [30]. The ${\hat{p}}^{1}$ and ${\hat{p}}^{0}$ frequencies are

$${\hat{p}}^{1}=\frac{1}{P}\sum_{j=1}^{P}\frac{w_{j}}{\sum_{j=1}^{P}w_{j}}\frac{m_{j}^{1}}{{2N}^{1}}\;{\hat{p}}^{0}=\frac{1}{P}\sum_{j=1}^{P}\frac{w_{j}}{\sum_{j=1}^{P}w_{j}}\frac{m_{j}^{0}}{{2N}^{0}}$$

with *w*_*j*_ the weight for the *j*-th variant. DoEstRare use a similar ponderation approach than the KBAC test. It assumes that the count of rare mutations in cases $M_{j}^{1}$ follows, under the null hypothesis, a binomial distribution $B\left(2N^{1},{\hat{MAF_{j}}}^{0}\right)$ with ${\hat{MAF_{j}}}^{0}$ the estimate of the minor allele frequency in controls. The weight *w*_*j*_ is the probability to present less than the observed count $m_{j}^{1}$.

$$w_{j}=P(M_{j}^{1}\leq m_{j}^{1})=\sum_{k=0}^{m_{j}^{1}}(\begin{array}{c} 2N^{1}\\ k \end{array}){\left({\hat{MAF_{j}}}^{0}\right)}^{k}{\left(1-{\hat{MAF_{j}}}^{0}\right)}^{2N^{1}-k}$$

Other statistical tests [31–33,28,29] take into account position information in the test statistic but they were not taken into account in our comparison.

#### Weighting systems

Some of the statistical association tests we presented above use a weighting system to better discriminate causal from neutral variants. wSum and SKAT tests use of a function of the MAF estimate in the dataset. Because allele frequencies differ between populations, the computation of the MAF in the dataset will depend on the geographical origin of cases and controls. As we wanted to assess the impact of different weighting systems based on MAF estimation in the context of population stratification, we considered three weighting systems for both Sum and SKAT tests:

1. an unweighted version with *w*_*j*_ = 1, *j* ∈ {1,…,*P*} (labeled Sum, SKAT and SKATO);
2. weights following a beta distribution $w_{j}=Beta(\hat{MAF_{j}},a_{1}=1,a_{2}=25)$, as proposed by [9], with $\hat{MAF_{j}}$ the MAF estimation in both cases and controls (labeled wSum_betaMAFtot, wSKAT_betaMAFtot and wSKATO_beta_MATtot);
3. weights $w_{j}=\frac{1}{\sqrt{N*\hat{MAF_{j}}*(1-\hat{MAF_{j}})}}$, as proposed by [3], but with the MAF estimation in both cases and controls (labeled wSum_MAFtot, wSKAT_MAFtot, wSKATO_MAFtot).

We also considered, for the Sum test, a fourth weighting system $w_{j}=\frac{1}{\sqrt{N*{{\hat{MAF}}_{j}}^{0}*(1-{{\hat{MAF}}_{j}}^{0})}}$, from [3], with ${{\hat{MAF}}_{j}}^{0}$ the MAF estimation in controls (labeled wSum_MAFctrl). This weighting system cannot be used in the SKAT R Package, as it would introduce a bias without a proper permutation procedure.

### Simulation workflow

**Fig 1:**
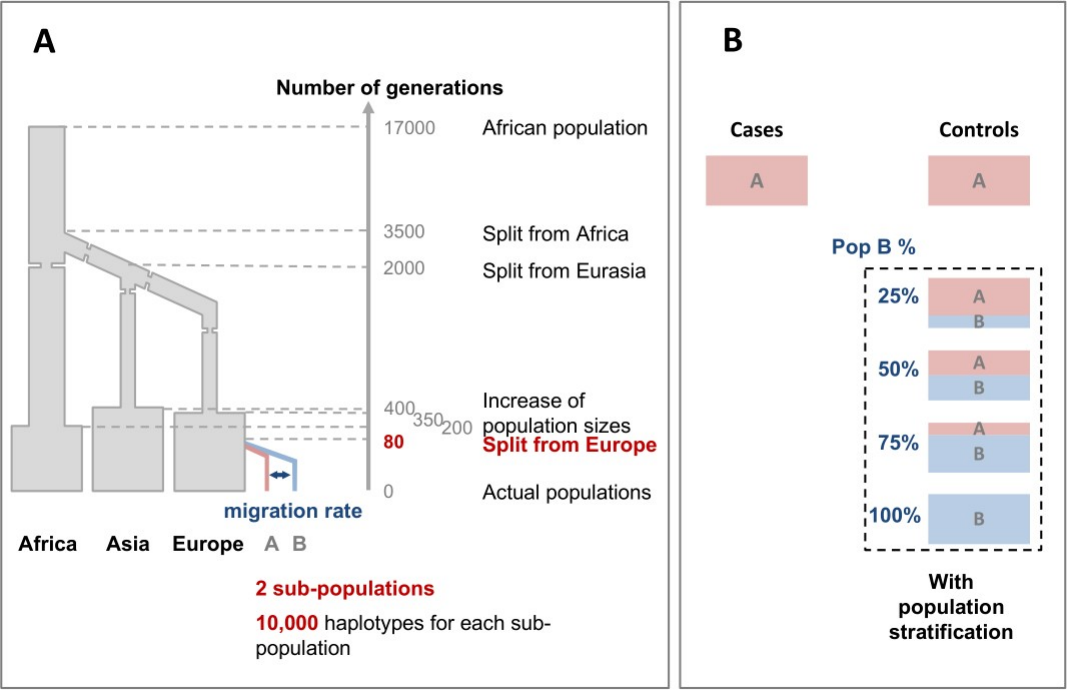
Simulation of a population stratification in case-control data. A. Simulated demographical model with the *cosi* program. Modifications from the bestfit model designed by [34] are indicated in red. The migration parameter we varied is in dark blue. B. Geographical origin of cases and controls. In a first scenario cases and controls are from the same population A. In scenarios with a population stratification, the percentage of controls from population B we varied is indicated in dark blue.

We used the program *cosi* [34], based on a coalescent model, to simulate genetic data. The coalescent model’s demographical parameters are derived from the bestfit model which was found to best explain present worldwide genetic diversity [34]. We added two European subpopulations A and B, which split from the original European population 80 generations ago (**Fig 1A**). Each sub-population includes 10,000 haplotypes (5,000 individuals). The geographical proximity between these two populations is linked to the migration rate parameter. This migration rate parameter varies between 0, 0.001, 0.01, 0.025, 0.05 and 0.1, to investigate the impact of population structure at different geographical scales.

From the two sub-populations, we sampled 1,000 cases and 1,000 controls according different genetic scenarios (**Fig 1B**). In all scenarios, cases are from the same population A, and controls from populations A and B. The proportion of controls coming from the population B varies between 25%, 50%, 75% and 100%. We also simulated a scenario without population stratification with all cases and controls from population A.

These scenarios varying the population structure are simulated under the *H*_0_ (no genetic association) and *H*_1_ (genetic association) hypotheses in order to assess respectively type I error and power.

Under the null hypothesis, cases and controls are sampled from the two populations A and B without regarding the genetic information.

Under the alternative hypothesis, the disease status is sampled with the probability *P*(*Y*_*i*_ = 1|***X***_***i***_) determined by the following logistic regression model:

$$logit(P(Y_{i}=1|X_{i}))=\alpha_{0}+\beta^{\prime }X_{i}$$

with ***β***′ the vector of genetic regression coefficients. We considered in this model, that 50% of rare variants are deleterious with an odd ratio *OR* = 1.5. The individual sampling process is repeated until obtaining 1,000 cases and 1,000 controls with a given percentage of controls from population B.

Rare variants are defined according to the MAF in the total population A (10,000 haplotypes) as cases are sampled from this population A.

We performed 10,000 replicates to assess type I errors and 1,000 replicates to assess power.

### Exploratory analysis of simulated data

We explored our simulated data to assess how close are the populations A and B, by (1) performing PCA, and (2) computing the fixation index F_ST_ [35] that measures the population differentiation due to genetic structure. We applied these methods to the genetic data concatenating all common genetic variants from the 10,000 gene replicates. Genetic data include pruned common variants with a *MAF* ≥ 5% and *r*^2^ < 0.2 in the total population A, for a sampling of 1,000 individuals in each population A and B.

We performed the PCA with the *smartpca* program from EIGENSOFT package version 6.1.4 [36,37]. We computed the fixation index F_ST_ between populations A and B using the R function *calc_wcFst_spop_pairs* from the github repository https://github.com/ekfchan/evachan.org-Rscripts, which implements the method of [35].

### Rare variant association analysis and population stratification correction

Analyses of the association of rare variants are carried out using the statistical tests previously described, on data simulated according to different scenarios mentioned above. Significance is assessed with an adaptive permutation procedure [38] for all statistical tests except SKATs and PODKAT, which rely on a approximated distribution of the test statistic under the null hypothesis. The adaptive permutation procedure enables to save computational time in comparison with the standard permutation procedure. Parameters are the significance threshold *α* = 0.01 and the precision value *c* = 0.2. Power and type I error have been assessed considering α = 0.05.

Statistical tests are performed with and without correcting for population structure. The most common correction method is the integration of covariates, such as PCs computed from the genetic data, in a logistic regression model [37]. As it is described before in this paper, most of statistical tests, with the exception of KBAC and DoEstRare, are presented under the form of a logistic regression model and can be adjusted with this method.

$$M_{1}\;logit(P(Y_{i}=1|X_{i},Z_{i}))=\alpha_{0}+\alpha^{\prime }Z_{i}+\beta^{\prime }X_{i}$$

with ***Z***_***i***_ the vector of covariates for the *i*-th individual. We label this method “PCA model correction”.

Note: As it is mentioned by authors of KBAC, this test can be adjusted with covariates in a logistic regression model but is not implemented in the R package.

Another correction method, proposed by [39], using a permutation procedure taking into account covariates, can be applied to a larger range of association tests. First, the null model is adjusted for covariates:

$$M_{0}\;logit(P(Y_{i}=1|,Z_{i}))=\alpha_{0}+\alpha^{\prime }Z_{i}$$

Then, from this model, odds of disease conditional on covariates *θ*_*i*_ = exp(*P*(*Y*_*i*_ = 1|,***Z***_*i*_)),*i* ∈ {1,…,*N*} are computed. Finally, individuals are sampled according to a Fisher’s noncentral hypergeometric distribution with disease odds *θ*_*i*_ as individual weights, to obtain permutated data with similar population stratification. We label this method “PCA permutation correction”. For significance assessment, we adapted the adaptive permutation procedure we used [38], to take into account PCs, according to the description of [39], on all statistical tests including SKATs and PODKAT. Because the permutation procedure is running very slowly for SKATs and PODKAT, we assessed only type I errors with this correction method for a number of 5,000 replicates instead of 10,000.

These two correction methods rely on covariates, reflecting the geographical origin of individuals. We considered the two first components of the PCA performed with *smartpca* program from EIGENSOFT package version 6.1.4 [36,37]. PCA was performed on pruned common variants (*MAF* ≥ 5% and *r*^2^ < 0.2 in the total population A), for each case-control sampling according to the genetic scenario. We also performed type I error analyses with 5 PCs and 10 PCs on a subset of 5,000 replicates instead of 10,000 to assess the consequences of the number of PCs setting.

## Results and discussion

### Simulation of a fine geographical scale population structure

**Fig 2:**
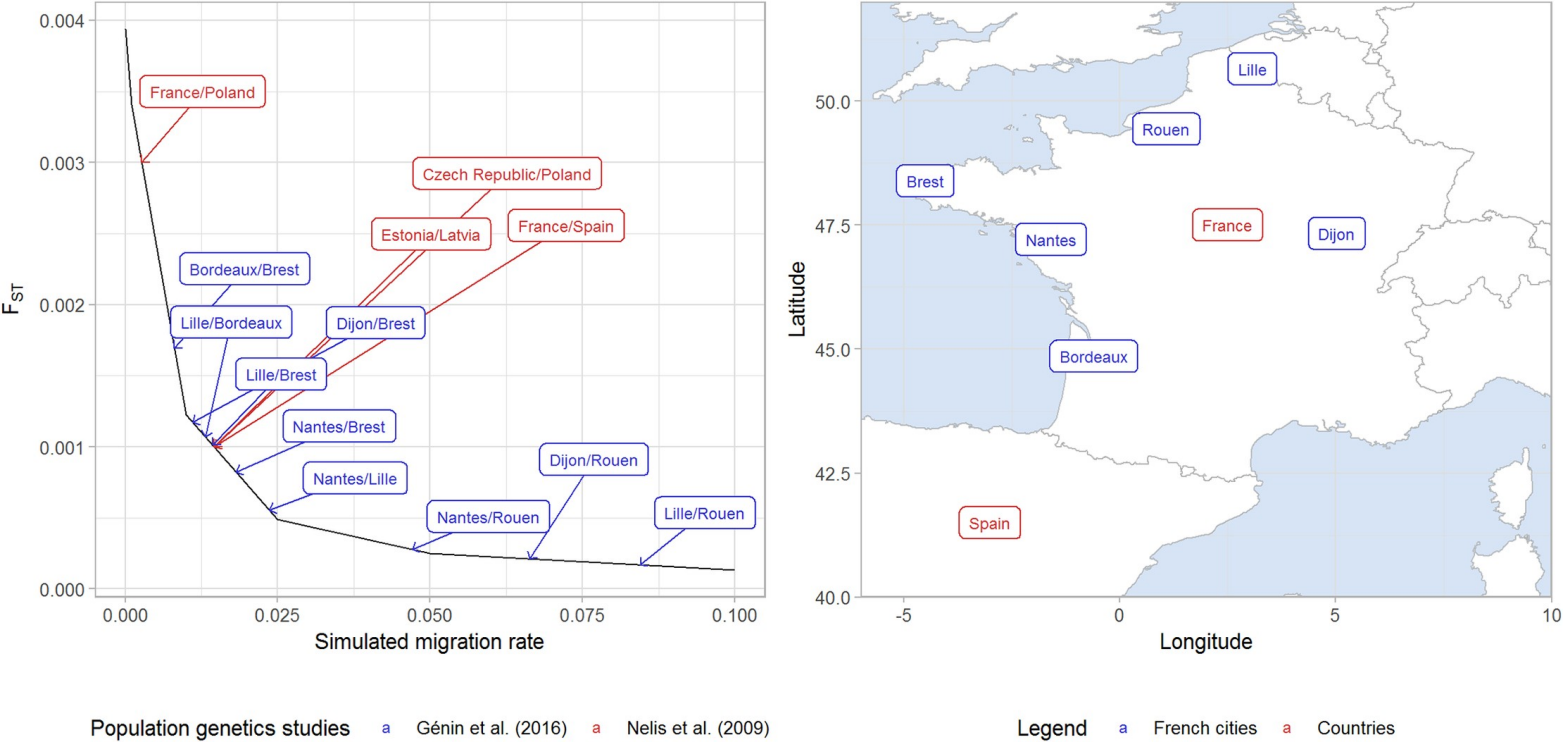
Comparison of F_ST_ values between simulations and real population genetic studies. FST values obtained from simulations are plotted in function of the migration rate parameter. Pairwise FST values from two real population genetic studies, [40] and [41], are added respectively in red and blue.

We simulated genetic information for 10,000 artificial genes for two populations A and B varying the migration rate parameter between 0, 0.001, 0.01, 0.025, 0.05 and 0.1. Numbers of SNV and allele frequency distributions for 1,000 individuals are almost the same in populations A and B (**Table A** and **Table B** in **S1 Table**). Depending on the migration rate, the number of SNV varies between 702,332 and 846,053 (**Table A** in **S1 Table**), and the percentage of rare variants with a MAF< = 1% varies between 53.5% and 61.7% (**Table B** in **S1 Table**) in population A. In order to assess the geographical differentiation level of populations A and B for each migration rate, we computed pairwise F_ST_ values and performed PCA on pruned common variants. In the **Fig 2** (see **Table C** in **S1 Table** for F_ST_ values), we related these values to estimates from [40] and from [41], which are respectively genetic studies on European populations and the French population. In [40], neighbor European countries present F_ST_ values close to 0.001 (France/Spain: 0.001; Czech Republic/Poland: 0.001; Estonia/Latvia: 0.001). Far European countries show higher F_ST_ values (France/Latvia: 0.008, Latvia/Spain: 0.01). Our simulations with a migration rate of 0.01 conduct to a F_ST_ value close to 0.001 (F_ST_(0.01) = 0.001226), which would correspond to a situation with neighbor countries. Simulations with migration rates of 0 and 0.001 correspond to situations with more distant countries. Scenarios that correspond to fine-scale population structure, are scenarios with a migration rate higher than 0.01. In [41], results are from the French Exome project (FREX)., in which controls are recruited from 6 French centers (Bordeaux, Brest, Dijon, Lille, Nantes, and Rouen cities) and had their exome sequenced. The scenario with a migration rate of 0.001 shows a F_ST_ value also close to the estimates for distant French regions. Scenarios with a migration rate of 0.025, 0.05 or 0.1 would correspond to situations with geographically close French regions, which means a very fine-scale population structure. Of course, this “inference” is based on only the F_ST_ indicator, and other parameters should be taken into account such as allele frequency distributions.

We also perform a PCA analysis on simulated data, to see whether it is possible to distinguish the populations A and B from individual genetic profiles. The representations of the two first components (**S1 Fig**) show that populations are clearly distinct with a migration rate≤0.01. The overlaps of genetic profiles between populations A and B are respectively very small, moderate, and nearly total for scenarios with a migration rate equal to 0.025, 0.05 and 0.1. In the PCA analysis of FREX data from Génin et al. (2016), not presented here, French sub-populations showed moderate to high overlaps. However, we cannot compare their PCA results with ours as sampling sizes are very different: around 100 individuals per French sub-population while 1,000 individuals per simulated population.

### Inflation of type I errors and efficiency of correction methods

Cases and controls, for the rare variant association analysis, are sampled from the two simulated populations A and B. We conducted simulations under *H*_0_ to assess type I errors. For 2,000 individuals from the population A, the average number of analyzed SNV, across the 10,000 replicates, varies between 30.8 (se: 7.3) and 32.5 (se: 7.5) depending on the migration rate (respectively 0.01 and 0.1). Without population stratification (case and all controls coming from population A), type I errors at significance level α = 5% are correct with the exception of CAST which seems conservative (**S2 Table** and **S2 Fig**).

**Fig 3:**
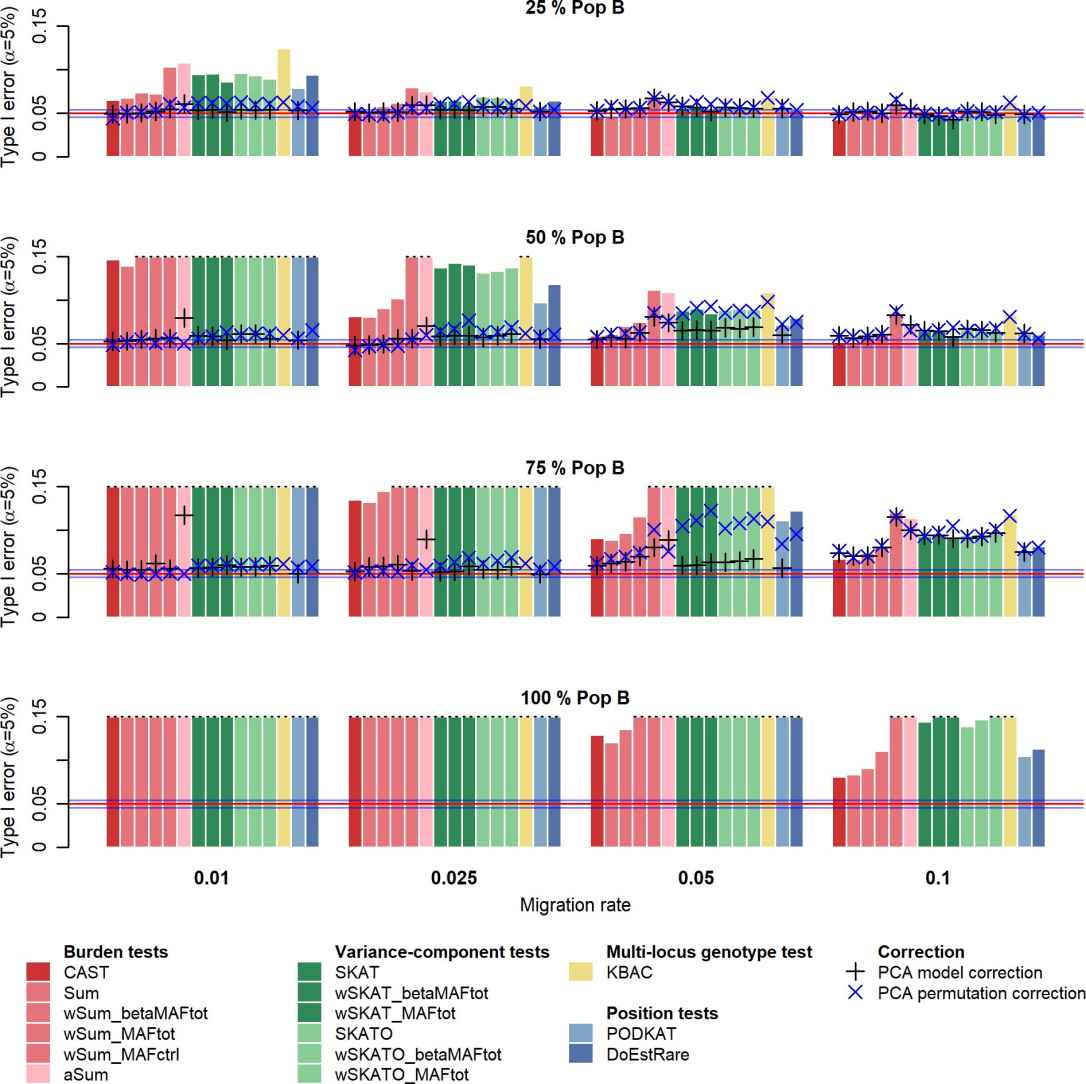
Type I errors at level α = 5% with a population stratification. Bars represent type I errors without correction for population stratification. The red line corresponds to α = 5% and blue lines correspond to 95% confidence interval. Confidence interval is computed assuming that the number of false positives follows a binomial distribution with parameters 10,000 and 0.05. Correction methods are performed with the first two PCs.

We then analyzed simulated datasets with mixed ancestry in controls, first without any correction method, in order to estimate the increase of type I errors due to population stratification. Type I errors show inflation even in the presence of a very fine scale population structure, i.e. with a migration rate of 0.05 or 0.1 (**Fig 3**). However, this inflation remains negligible when 25% or less of controls are ascertained from population B, which means that cases and controls are still quite homogenous in terms of geographical origin.

We note obvious type I error differences between rare variant association tests. CAST and Sum burden tests are the least sensitive to population stratification in almost all scenarios, while the wSum_MAFctrl and aSum burden tests, which are just variations of the previous test, present high values of inflation. Variance-component tests and KBAC present high type I errors compared to other tests. Finally, tests integrating variant positions present intermediate values. These results are consistent with observations made by [18] and [15] where variance-component tests also presented higher inflation than some burden tests. From all these observations, it is understandable that variance-component tests and the aSum test present higher type I error values as they are adapted to test a group of rare variants with opposed effects (protective/risk variants). Indeed, both populations A and B include population-specific rare variants, in the sense that some rare variants are more frequent in one population. This creates a situation where rare variants seem to display opposed effects, when cases and controls are sampled from two populations. However, it has been discussed by [18] the possibility of burden tests being more sensitive than variance-component tests if global count of rare variants differ between populations, and may be influenced by demographical conditions such as population growth. We did not consider different growth rates in population A and B, also explaining why variance-component tests are more sensitive.

Weighting allele contribution according to MAF is common practice in rare variants tests, based on the assumption that the probability of functional, usually harmful, effect is increased for very rare alleles. The weighted derived version of Sum test, wSum_MAFctrl, which uses MAF the estimation in controls, presents a very high inflation of type I errors. When MAF is estimated in both controls and cases, wSum_MAFtot and wSum_betaMAFtot also present a higher inflation, but a lot lower in comparison with wSum_MAFctrl. The test wSum_MAFtot seems to present a slight increase of type I error compared to wSum_betaMAFtot. These two tests differ in the MAF weighting function, which is standard deviation or beta distribution. By using a beta distribution of parameters 1 and 25, in wSum_betaMAFtot, weights decrease less rapidly with the MAF increase and may thus buffer the effect of wrong MAF weighting. This difference induced by using beta weights is less clear for variance-component tests, wSKAT_MAFtot and wSKATO_betaMAFtot, as it is only visible with high proportions of controls from population B and low migration rates, i.e. with the highest population stratifications (see **S2 Table**). We conclude that the use of a weighting system based on the computation of MAFs, from the dataset, may provide high numbers of false positives when allele frequencies differ between cases and controls due to stratification. We can also extend this interpretation to the KBAC and the DoEstRare tests, as weighting systems depend on observed allele counts in controls.

In practice, a correction method can and should be applied to avoid statistical biases from the population stratification. We applied two correction methods, the “PCA model correction” and the “PCA permutation correction”, integrating the information from the two first PCs on common variants from the 10,000 replicates, to reduce the inflation of type I errors. In the scenarios with 100% of controls from population B, these two correction methods were not applied as PCs may totally explain the phenotype, not allowing testing for genetic effects. These correction methods perform well in scenarios with the most differentiated populations (migration rate of 0.01); but they do not totally reduce the inflation caused by a very fine scale population structure (migration rate of 0.05 or 0.1). The “PCA permutation correction” is advantageous with association tests which are not based on a logistic regression model, but seems to be less efficient in the scenario with a migration rate of 0.05. It suggests that the “PCA permutation correction method” gradually lose more in efficiency than the “PCA model correction method” with finer population stratification.

We integrated the information from the two first PCs, as it was sufficient to correct with the largest population structures. As in practice the choice of the number of PCs to integrate in the models may be arbitrary and adapted depending on the scenario, we here performed analyses by integrating 2, 5 or 10 PCs (**S2 Table** and **S3 Fig**). By increasing the number of PCs in the models, the type I error inflation does not decrease in the context of very fine scale population structures (migration rate of 0.05 or 0.1), hence our choice to keep only two PCs. We even note an increase of type I errors in some scenarios for burden tests, whose significance is assessed by an adaptive permutation procedure. It is maybe due to an over-adjustment of the logistic regression model with a high number of PCs [42].

Our analyses were conducted considering a small sample size of 1,000 cases and 1,000 controls. By increasing the sample size, type I errors may increase greatly (see S4 Fig). A fine population structure in the data would have even more impact in large sample sequencing studies.

Type I errors might also differ considering other demographical models resulting in very different site frequency spectrum [43–45]. In our simulations, we used a derived model from [34], in which the population expansion events are instantaneous. However, this model is very simplistic and does not reflect demographical growth observations.

In this study, type I errors have been assessed considering α = 0.05. We realize that the actual significance threshold being used in genetic studies is much lower after correcting multiple testing (α = 2.5e-6 when considering 20,000 genes in an exome-sequencing study). Test statistics may behave differently at very low significance levels but because a large subset of our tests is using time-consuming permutations, whose number could not be increased within the scope of the present study.

### Balance between type I error and power under population stratification

**Fig 4:**
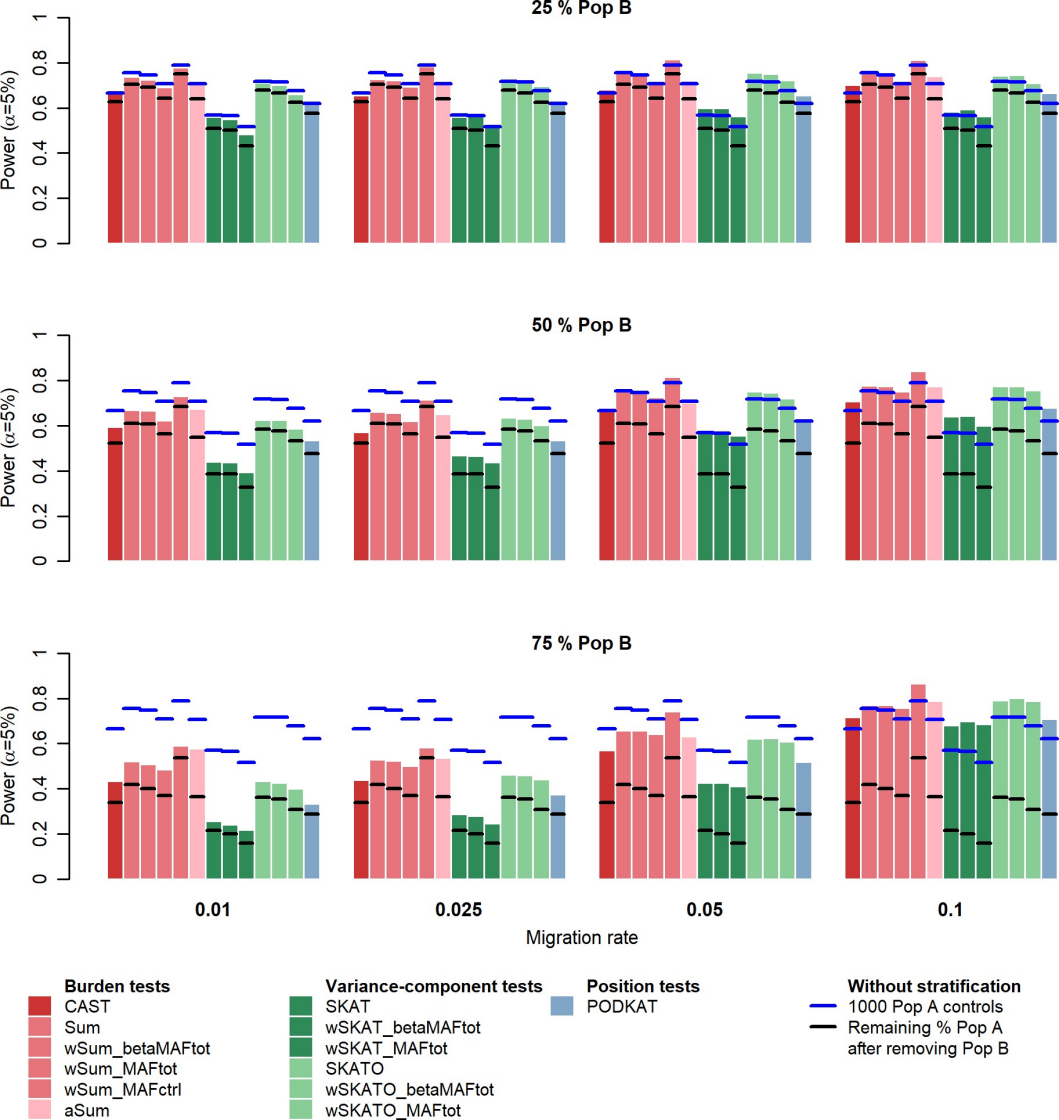
Powers at α = 5% after PCA model correction. PCA model correction was performed on all scenarios except when 100% of controls are from population B (geographical covariates may totally predict the phenotype).

The purpose of using external controls, i.e. from population B in our scenarios, is to increase the power to detect deleterious genes in association studies when it is impossible to sequence larger sample size of controls. We observed previously that stratification correction methods enable to reduce statistical biases with the largest population structures but fail with the finest ones. For this reason, we aim to assess the impact of the “PCA model correction” on the power of rare variant association tests. We simulated a simple scenario under *H*_1_, with half of rare variants being deleterious with an OR of 1.5. In the **Fig 4**, with the most structured simulated populations, i.e. with high percentages of controls from population B and low migration rates, we can observe an obvious loss of power for every statistical test, compared to the analysis without population stratification (see **S3 Table** for power values). In these scenarios, deleterious variants are likely to be under population structure, i.e. presenting different allele frequencies in populations A and B. The adjustment for PCs also removes, from rare variant association tests, a portion of the disease genetic component. For the finest population structures, we note a small increase of power with the use of controls from population B, due to statistical biases not fully (or at all) corrected.

We also estimated powers after removing from the analysis controls from the population B. For example, in the scenario with 75% controls from population B, only 25% controls are remaining in the analysis. Obviously, the power is decreased in these analyses as the number of controls is much less. More interestingly, with large scale population structures, we observe that the power is not much higher when adding controls from population B. As we discussed previously, the PCs capture the disease genetic component which is confounded with the genetic population structure. The power is, as expected, greatly increased when adding controls from a very close population. However, this advantage is balanced by the increase of type I error which is not fully corrected by adjusting the model.

## Conclusions

Our simulation study showed that, as expected, we observed a very high inflation of type I errors in the presence of strong stratification. This inflation could, in principle, be controlled by through standard correction methods. In this work, our objective was to assess the impact at a finer geographical scale, as rare variants tend to be more localized than common variants. In this context, the inflation was still present but notably smaller. Intriguingly, the standard methods were less efficient at correcting this bias, as they did not effectively capture geographical origin from genetic data. This work underlies the importance of selecting controls with similar genetic background even at very fine geographical scales in sequencing studies. Exploratory analyses of the population structure should not be neglected and adjusting for potential bias must be done carefully.

## Supporting information

S1 Fig PCA plots in function of the migration rate. PCA was performed on the pruned dataset (MAF≥5% and r^2^≤0.2 in the total population A) with 1,000 individuals from each population A and B.
(PNG)

S2 Fig Type I errors at α = 5% without population stratification. The red line corresponds to α = 5% and blue lines correspond to 95% confidence interval. Confidence interval is computed assuming that the number of false positives follows a binomial distribution with parameters 10,000 and 0.05.
(PNG)

S3 Fig Type I errors at α = 5% varying the number of PC to integrate in the PCA model correction method. The red line corresponds to α = 5% and blue lines correspond to 95% confidence interval. Confidence interval is computed assuming that the number of false positives follows a binomial distribution with parameters 10,000 and 0.05.
(PNG)

S4 Fig Type I errors at α = 5% varying the number of cases and controls. The red line corresponds to α = 5% and blue lines correspond to 95% confidence interval. Confidence interval is computed assuming that the number of false positives follows a binomial distribution with parameters 10,000 and 0.05.
(PNG)

S1 Table Supplementary tables from Table A to Table C. Table A: Number of SNV in populations A and B for 10,000 simulated genes; Table B: Variant frequency distributions in populations A and B for 10,000 simulated genes; Table C: FST values for simulated data in function of the migration rate.
(DOCX)

S2 Table Type I error values at α = 5%. (CSV)

S3 Table Power values at α = 5%. (CSV)
