# Supplementary figures and images for: The impact of a fine-scale population stratification on rare variant association test results
Source: PLoS One. 2018 Dec 6;13(12):e0207677. doi: 10.1371/journal.pone.0207677 (PMC6283567; doi:10.1371/journal.pone.0207677)

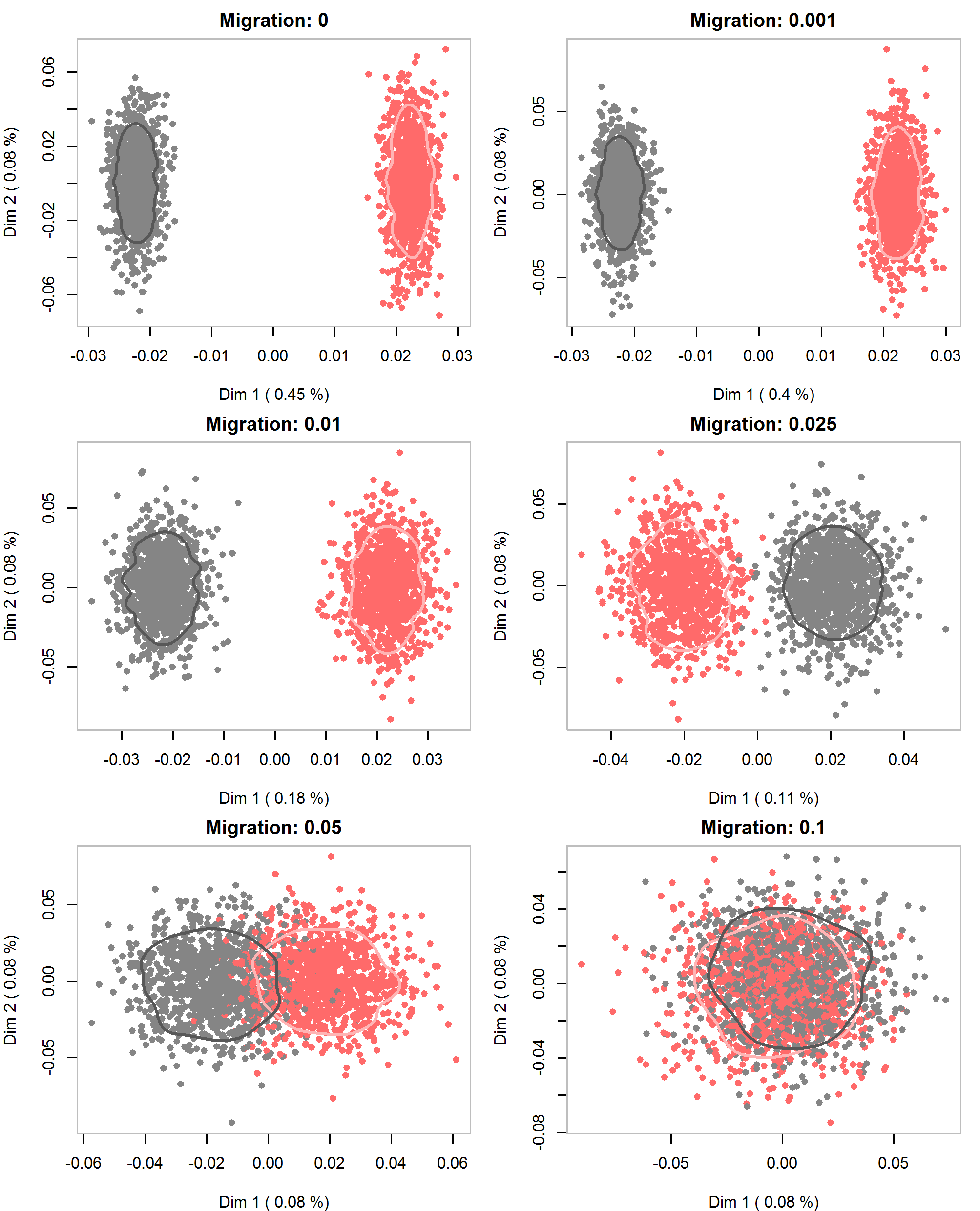

Supplement: S1 Fig — PCA was performed on the pruned dataset (MAF≥5% and r2≤0.2 in the total population A) with 1,000 individuals from each population A and B. (PNG) [file pone.0207677.s001.png]

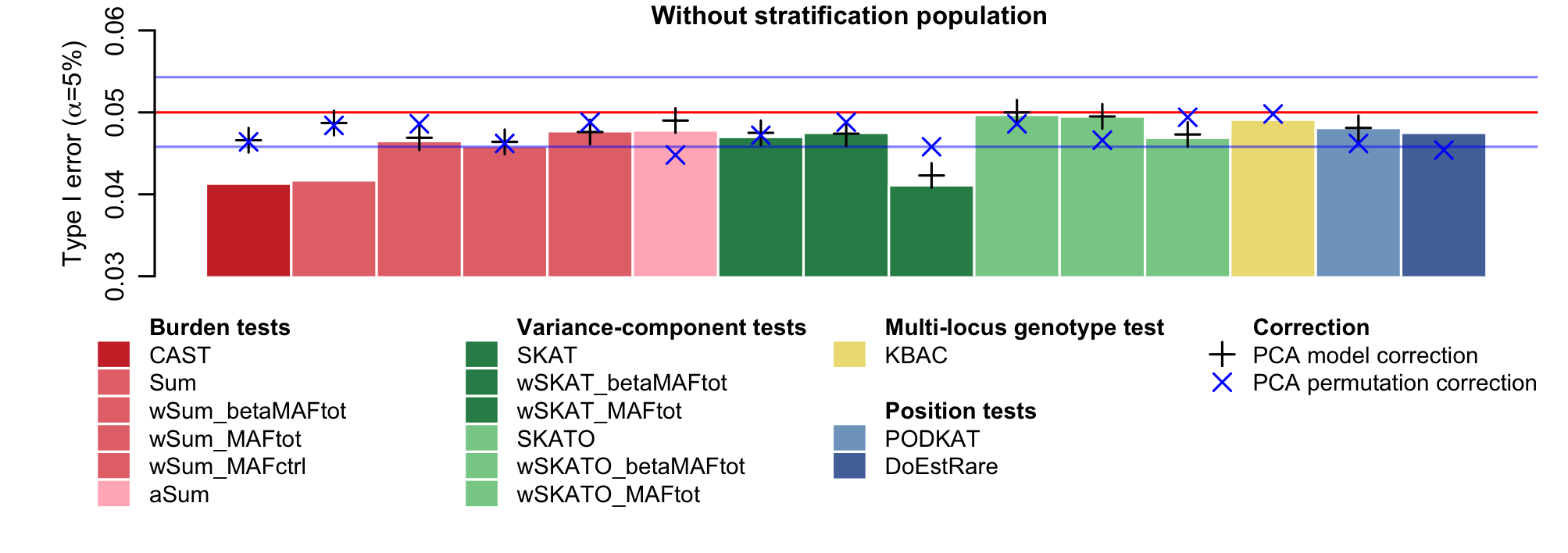

Supplement: S2 Fig — The red line corresponds to α = 5% and blue lines correspond to 95% confidence interval. Confidence interval is computed assuming that the number of false positives follows a binomial distribution with parameters 10,000 and 0.05. (PNG) [file pone.0207677.s002.png]

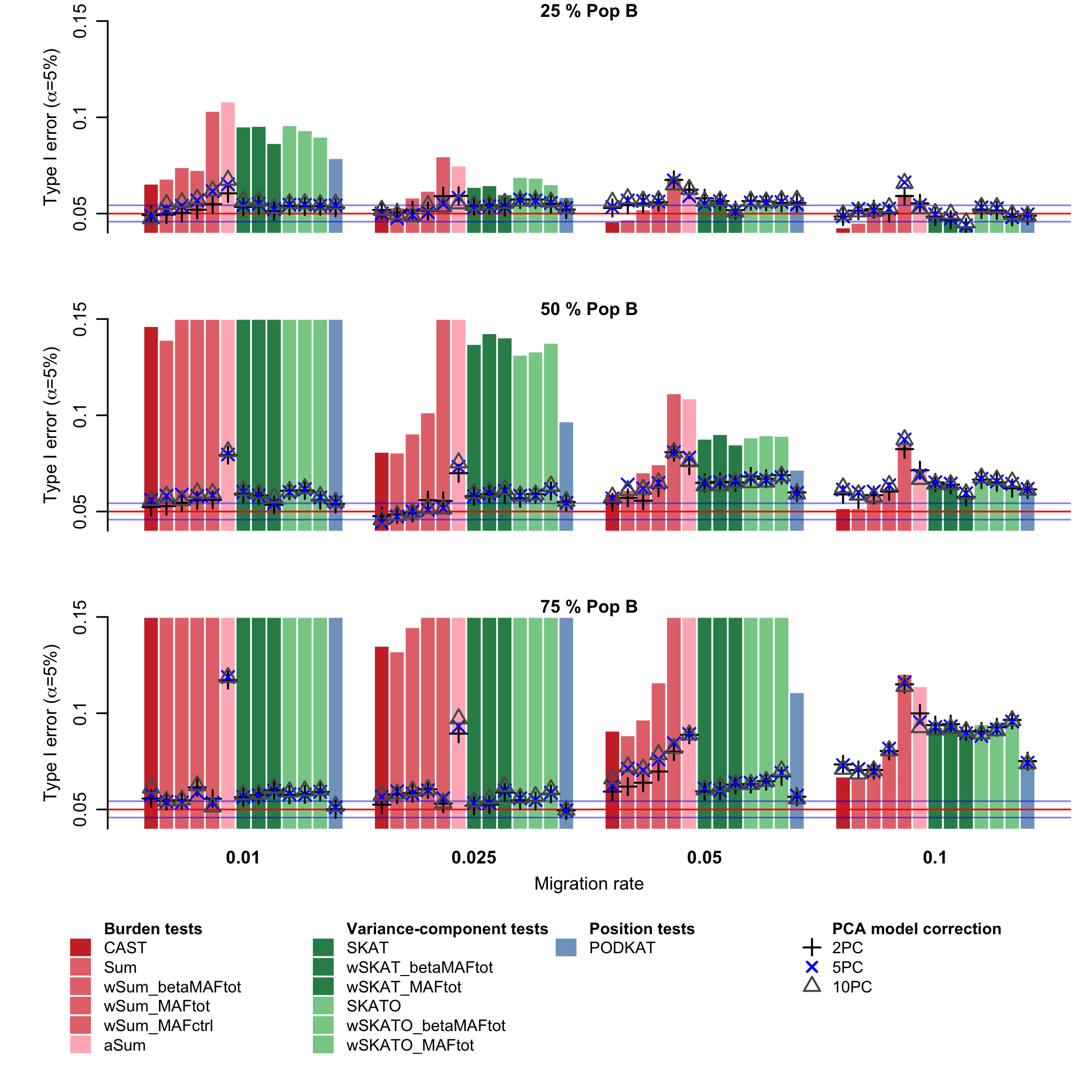

Supplement: S3 Fig — The red line corresponds to α = 5% and blue lines correspond to 95% confidence interval. Confidence interval is computed assuming that the number of false positives follows a binomial distribution with parameters 10,000 and 0.05. (PNG) [file pone.0207677.s003.png]

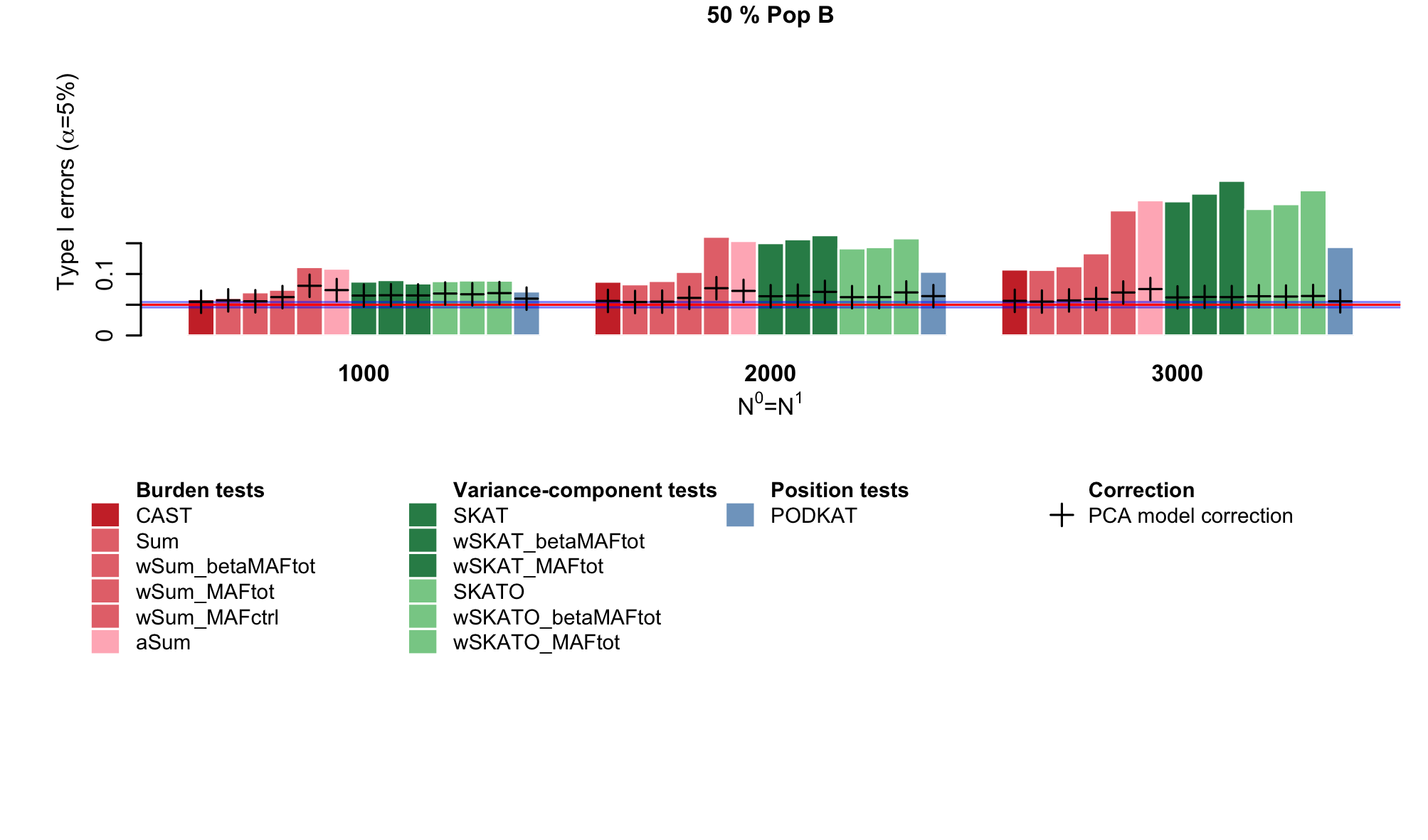

Supplement: S4 Fig — The red line corresponds to α = 5% and blue lines correspond to 95% confidence interval. Confidence interval is computed assuming that the number of false positives follows a binomial distribution with parameters 10,000 and 0.05. (PNG) [file pone.0207677.s004.png]
