# Supplementary material for: The impact of a fine-scale population stratification on rare variant association test results
Source: PLoS One. 2018 Dec 6;13(12):e0207677. doi: 10.1371/journal.pone.0207677 (PMC6283567; doi:10.1371/journal.pone.0207677)
Supplement: S1 Table — Table A: Number of SNV in populations A and B for 10,000 simulated genes; Table B: Variant frequency distributions in populations A and B for 10,000 simulated genes; Table C: FST values for simulated data in function of the migration rate. (DOCX) [file pone.0207677.s005.docx]

**Table A. Number of SNV in populations A and B for 10,000 simulated genes.**

| **Migration rate** | **Pop A (N=1,000)** | **Pop B (N=1,000)** |
| --- | --- | --- |
| 0 | 702332 | 701915 |
| 0.001 | 725467 | 724992 |
| 0.01 | 799779 | 799471 |
| 0.025 | 824737 | 824405 |
| 0.05 | 839808 | 839490 |
| 0.1 | 846053 | 846555 |

**Table B. Variant frequency distributions in populations A and B for 10,000 simulated genes.**

| **Migration rate** | **MAF range** | **Pop A (N=1,000)** | **Pop B (N=1,000)** |
| --- | --- | --- | --- |
| 0 | $\left[ 0.5, 0.05 \right[$ | 0.316 | 0.316 |
|  | $\left[ 0.05, 0.01 \right[$ | 0.149 | 0.149 |
|  | $\left[ 0.01, 0.001 \right[$ | 0.334 | 0.334 |
|  | $\left[ 0.001, 0 \right[$ | 0.201 | 0.201 |
| 0.001 | $\left[ 0.10, 0.05 \right[$ | 0.306 | 0.307 |
|  | $\left[ 0.05, 0.01 \right[$ | 0.143 | 0.143 |
|  | $\left[ 0.01, 0.001 \right[$ | 0.331 | 0.332 |
|  | $\left[ 0.001, 0 \right[$ | 0.219 | 0.219 |
| 0.01 | $\left[ 0.10, 0.05 \right[$ | 0.278 | 0.278 |
|  | $\left[ 0.05, 0.01 \right[$ | 0.129 | 0.130 |
|  | $\left[ 0.01, 0.001 \right[$ | 0.327 | 0.327 |
|  | $\left[ 0.001, 0 \right[$ | 0.266 | 0.265 |
| 0.025 | $\left[ 0.10, 0.05 \right[$ | 0.268 | 0.269 |
|  | $\left[ 0.05, 0.01 \right[$ | 0.123 | 0.124 |
|  | $\left[ 0.01, 0.001 \right[$ | 0.327 | 0.327 |
|  | $\left[ 0.001, 0 \right[$ | 0.281 | 0.281 |
| 0.05 | $\left[ 0.10, 0.05 \right[$ | 0.265 | 0.265 |
|  | $\left[ 0.05, 0.01 \right[$ | 0.122 | 0.122 |
|  | $\left[ 0.01, 0.001 \right[$ | 0.325 | 0.325 |
|  | $\left[ 0.001, 0 \right[$ | 0.287 | 0.288 |
| 0.1 | $\left[ 0.10, 0.05 \right[$ | 0.262 | 0.262 |
|  | $\left[ 0.05, 0.01 \right[$ | 0.120 | 0.120 |
|  | $\left[ 0.01, 0.001 \right[$ | 0.323 | 0.323 |
|  | $\left[ 0.001, 0 \right[$ | 0.294 | 0.295 |

**Table C. F_ST_ values for simulated data in function of the migration rate.**

| **Migration rate** | **Number of variants** | **F_ST_** |
| --- | --- | --- |
| 0 | 37,449 | 0.003940 |
| 0.001 | 37,532 | 0.003410 |
| 0.01 | 37,583 | 0.001226 |
| 0.025 | 37,294 | 0.000487 |
| 0.05 | 37,718 | 0.000251 |
| 0.1 | 37,561 | 0.000132 |
